# Years of life lost due to gastric cancer is increased after Bayesian correcting for misclassification in Iranian population 

**Published:** 2016

**Authors:** Nastaran Hajizadeh, Mohamad Amin Pourhoseingholi, Ahmad Reza Baghestani, Alireza Abadi, Behnaz Ghoreshi

**Affiliations:** 1 *Department of Biostatistics, Shahid Beheshti University of Medical Sciences, Tehran, Iran*; 2 *Gastroenterology and Liver diseases Research Center, Research Institute for Gastroenterology and Liver diseases, Shahid Beheshti University of Medical Sciences, Tehran, Iran *; 3 *Faculty of Medicine, Islamic Azad University, Tehran, Iran *

**Keywords:** Misclassification, Bayesian method, Years of life lost, Gastric cancer, Iran

## Abstract

**Aim:**

To estimate the change in years of life lost (YLL) due to gastric cancer mortality after correcting for misclassification in registering causes of death using the Bayesian method.

**Background:**

For evaluating the health status of a country and determining priority of risk factors, some epidemiologic indicators are needed. Due to premature death, YLL is one of the most widely used indicators. To have an exact estimate of YLL, an accurate death registry data is needed, but the Iranian death registry is subject to misclassification error.

**Material and methods:**

Gastric cancer mortality data from 2006 to 2010 for Iran were extracted from national death statistics. The rate of misclassification in registered causes of deaths was estimated, using Bayesian method for each year. Then YLL of gastric cancer is estimated for different age-sex categories before and after implementing Bayesian method.

**Results:**

Using Bayesian method, the estimated misclassification rate for gastric cancer in cancer without label group were 5%, 3%, 3%, 7% and 7% respectively from 2006 to 2010. Estimated Years of life lost due to gastric cancer before correcting misclassification were respectively 111684.93, 114957.31, 112391.93, 112250.53 and 113300.92 person-years for years 2006 to 2010. After correcting misclassification, the total YLL of gastric cancer increased to 1535.19, 921.11, 908.39, 2566.39 and 2507.00 person-years, respectively from 2006 to 2010.

**Conclusion:**

If health policy makers ignore the existence of misclassification in registered causes of death, they may underestimate the burden of some causes of death and overestimate some others.

## Introduction

 Death statistics data are important to monitor the effects of screening programs, earlier diagnosis and other prognostic factors and can be used to guide policy makers to set up cancer prevention programs. With the increasing proportion of the population that has reached to the middle age and old age, the epidemiologic situation of developing countries has increasingly reflected adults’ diseases rather than that for children. Such a change in the demographical process and disease condition is commonly called epidemiologic transition (1). The investigation about the cause of death in Iran indicates that mortality due to contagious diseases has been declined. However, the mortality due to non-contagious diseases, particularly unintentional injuries, cardiovascular disease and cancer has been increased (2-4). 

Measures of mortality, such as age-specific death rates, life expectancy, cause-specific death rates and years of life lost (YLL), are commonly used to measure the health status of a population and are essential for epidemiological research and priority setting for health development (5-8). YLL is one of the most important indicators of prioritization in public health, which is an estimate of the average years a person would have lived if he or she had not died prematurely (9); and can be used as a basis for planning and evaluation of programs and interventions for prevention and control of diseases and their risk factors and also assessing the importance of different causes of death (10, 11). Accurate and reliable information on causes of death is one of the fundamental principles of planning, management and evaluation for health policy makers. In the death registration systems, especially in developing countries, including Iran, there are problems about registered data in terms of under reporting and misclassification of causes of death (12, 13). Misclassification error makes the registered data inaccurate and unreliable, leading to major problems in epidemiological analysis with biased estimates of burden, and underestimating the health risks (14, 15). According to the Iranian death registry, about 15% to 20% of death statistics are recorded in misclassified categories such as septicemia, cancer without mention of details, and other ill-defined conditions. Two statistical approaches are recommended to deal with misclassification error; first is using a small validation sample (16) and the second is Bayesian analysis, which provides subjective prior information for the subset of the parameters to correct the statistics (17). 

Cancer is the third most common cause of death in Iran (18) and among all malignancies, gastric cancer is a fatal one (19). The purpose of this study is to estimate the misclassification rate of gastric cancer which registered as cancer without mentioning detail, using a Bayesian method and estimating the difference that misclassification error makes in the YLL of premature death due to this malignancy in Iranian population. 

## Material and Methods

National death statistic from 2006 to 2010 for gastric cancer [ICD-10; C16] which reported annually by the Ministry of Health and Medical Education (MOH & ME) included in this study (20). This data set contains mortality data for all provinces other than Tehran for all years and Isfahan from 2007 to 2010.

To estimate the rate of misclassification of gastric cancer mortality in cancer without label group, a Bayesian approach was used with Poisson count regression based on the Pourhoseingholi, et al. approach (21, 22). To perform Bayesian inference, an informative beta prior distribution was assumed for the misclassified parameter. The initial value for the misclassified parameter is taken to be 20% based on the annual Iranian cancer registration reports. Because the misclassified parameter is unknown, a latent variable approach was employed to simplify the full conditional models and estimate the posterior distribution using a Gibbs sampling algorithm. 

For a group of deaths that occurred at ages within age interval *x *to *x *+ *n*, (i.e., *n *= age interval length), the expected years of life remaining for those deaths ($e_{n,x}^{s}$) is estimated using a formula for linear interpolation (Equation1) (11):

(1) $$e_{n,x}^{s}=e_{x}^{s}+\left(a_{n,x}-x\right)\frac{e_{x+n}^{s}-e_{x}^{s}}{\left(x+n\right)-x}$$

Where $a_{n,x}$ is the average age of death, and $e_{x}^{s}$ and $e_{x+n}^{s}$ are standard life expectancies based on theWest life table model, level 25 for male and 26 for female at ages *x *and *x *+ *n*, respectively. 

The crude expected years of life lost is (Equation 2):

$Y_{n,x}=\left(D_{n,x}\right)\left(e_{n,x}^{s}\right)$

(2)

Where $D_{n,x}$ is the number of deaths between age *x *and age *x *+ *n*.

Analyses were carried out using Excel and R software. 

## Results

There were 5029, 5691, 5564, 5557 and 5609 recorded deaths from gastric cancer, respectively, for years 2006 to 2010 in Iran cancer registry. The results of applying the Bayesian method show that there is 5% misclassification in 2006 in registering cause of death, as cancer while the underlying cause of death has been gastric cancer. This percent is estimated to be 3% for 2007 and 2008, as well as 7% for 2009 and 2010 based on the implemented Bayesian method. The estimated YLL for age categories for years 2006 to 2010, before and after correcting for misclassification error are shown in table 1 and 2 for men and women, respectively. The total YLL for men before the Bayesian correction of misclassification were calculated as 74610.58, 76796.67, 75082.88, 74988.42 and 75690.13 person-years and it increased to 75636.16, 77412.02, 75689.73, 76702.89, and 77364.92 person-years after Bayesian correction respectively for years 2006 to 2010. The total YLL for women before Bayesian correction of misclassification were calculated as 37074.35, 38160.63, 37309.04, 37262.11 and 37610.79 person-years and it increased to 37583.97, 38466.40, 37610.59, 38114.03 and 38443.00 person-years after Bayesian correction respectively for years 2006 to 2010. The total YLL for each year and the rate of that per 1000 were calculated by dividing YLL to the population and multiplying by 1000 to be compared with other countries. Also, the difference of YLL and the percent of change in YLL before and after the Bayesian correction of misclassification for each year are shown in table 3. Generally, YLL due to gastric cancer is increasing after correction for misclassification in registering underlying cause of death. 

## Discussion

Iran’s death registry is subject to misclassification in reporting underlying causes of death. A number of deaths due to gastric cancer are registered as cancer without mentioning the type of cancer. Using registered data with ignoring the existence of misclassification error leads to the underestimation of YLL of gastric cancer. 

In general, countries can be classified into two groups on the basis of availability of data on causes of death (7). One group is countries that have complete vital registration with medical certification of the cause of death assigned by physicians. The other group includes countries that have incomplete death registration systems, where causes of deaths are often recorded inaccurately at registration, resulting in large proportions of deaths assigned to ill-defined causes (5, 7).

**Table 1:** YLL of male due to gastric cancer mortality before and after Bayesian correction 2006-2010

Year	2006		2007		2008		2009		2010	
Age Group	Before	After	Before	After	Before	After	Before	After	Before	After
0-5	0	0	0	0	0	0	0	0	0	0
5-15	0	0	0	0	0	0	0	0	0	0
15-50	18514.37	18768.86	19056.84	19209.53	18631.57	18782.15	18608.13	19033.57	18782.25	19197.85
50-70	33812.23	34277.00	34802.93	35081.80	34026.27	34301.28	33983.46	34760.43	34301.47	35060.45
70+	22283.98	22590.29	22936.90	23120.69	22425.05	22606.29	22396.83	22908.89	22606.41	23106.62
Total YLL	74610.58	75636.16	76796.67	77412.02	75082.88	75689.73	74988.42	76702.89	75690.13	77364.92

**Table 2:** YLL of female due to gastric cancer mortality before and after Bayesian correction 2006-2010

Year	2006		2007		2008		2009		2010	
Age Group	Before	After	Before	After	Before	After	Before	After	Before	After
0-5	0	0	0	0	0	0	0	0	0	0
5-15	0	0	0	0	0	0	0	0	0	0
15-50	10951.80	11102.34	11272.68	11363.01	11021.12	11110.20	11007.26	11258.92	11110.26	11356.10
50-70	15057.71	15264.69	15498.90	15623.09	15153.03	15275.50	15133.97	15479.98	15275.59	15613.59
70+	11064.85	11216.94	11389.05	11480.30	11134.89	11224.88	11120.88	11375.14	11224.94	11473.32
Total YLL	37074.35	37583.97	38160.63	38466.40	37309.04	37610.59	37262.11	38114.03	37610.79	38443.00

**Table 3:** Total YLL of gastric cancer mortality before and after Bayesian correction and the difference 2006-2010

Year	Before	After	Difference	Percent of Change
2006	111684.93 (1.96)*	113220.12 (1.98)	1535.19	1.37
2007	114957.31 (2.06)	115878.42 (2.08)	921.11	0.80
2008	112391.93 (2.01)	113300.32 (2.02)	908.39	0.81
2009	112250.53 (1.97)	114816.92 (2.02)	2566.39	2.29
2010	113300.92 (1.95)	115807.92 (2.00)	2507.00	2.21

* Numbers in parentheses are rate of total YLL per 1000

The relative importance of gastric cancer among several important causes of mortality (from cancer and other diseases) can be evaluated by estimating the years of life lost (YLL). In a study in 2010-2011 with the aim of calculating the burden of 9 common cancers in the population covered by Mashhad University of Medical Sciences in Iran the most YLL was related to gastric, leukemia and lung cancers (1016, 9564 and 7061), respectively. In gastric cancer, 40% of years of life lost were due to premature death before age 60 (23, 24). The results of a study with the purpose of ranking the most important causes of death in terms of the number of Years of Life Lost (YLL) showed that unintentional injuries, cardiovascular diseases, diseases of the prenatal period, cancers and tumors, congenital and chromosomal malformations as well as diseases of the respiratory tract are the most important causes of years of life lost and these causes of death are responsible for about 82 percent of Iran’s YLL in 2006. The third cause of life lost after unintentional injuries and cardiovascular disease was cancers and tumors, which have decreased 420942 years of Iranians’ life in 2006. In that study, unintentional injuries, cardiovascular disease and cancer, were obtained as 27%, 25% and 10% for country’s YLL, respectively (25). Similar results were found in Naghavi. et al., study on mortality pattern in 29 provinces of Iran. They showed that 29%, 26% and 10% of YLL in the country in 2004 was due to unintentional injuries, cardiovascular disease and cancers, respectively (26). 

Studies on the burden of disease have considered accidents and traffic accidents on top and fewer studies have been performed on other major causes of death such as cardiovascular disease and cancers. This is despite the fact that cardiovascular disease and cancer have been the most common causes of death during the past decade. The consequences of the studies showed that the age of cardiovascular disease and cancer in the country has decreased in recent years. However, one might argue that everyone has to die of something eventually, and public health efforts should be directed at preventing premature death. When YLL is used as a measure of premature death, then injuries and infectious diseases become more important. While the most common cause of death for young people (aged 5 to 40) is injury and poisoning in the developed world, relatively few young people die and the principal causes of lost years remain cardiovascular disease and cancer (14). Since the cases of death that registered as cancer (without mentioning details) are usually excluded from calculations of YLL attributed to a special cancer, if such ill-defined codes redistributed to acceptable causes of deaths, YLL of a special cancer may even increase. 

Despite international efforts to facilitate and standardize processes for the collection and coding of data on causes of death, a significant proportion of deaths in the Islamic Republic of Iran misclassified to vague causes that can be reclassified to more specific causes of death. There is an urgent need to improve the quality of medical records and cause of death certification to have more accurate estimates of the burden of disease and consequently planning for disease control and prevention and allocation the resources.
